# Mental health and dual sensory loss in older adults: a systematic review

**DOI:** 10.3389/fnagi.2014.00083

**Published:** 2014-05-14

**Authors:** Chyrisse Heine, Colette J. Browning

**Affiliations:** ^1^Department of Human Communication Sciences, School of Allied Health, La Trobe UniversityBundoora, VIC, Australia; ^2^Primary Care Research Unit, School of Primary Health Care, Monash UniversityNotting Hill, VIC, Australia

**Keywords:** dual sensory loss, mental health, depression, systematic review

## Abstract

Mental health is a core component of quality of life in old age. Dual Sensory Loss (DSL; combined vision and hearing loss) is prevalent in older adults and has been correlated with decreased levels of well-being. This systematic review aimed to critically review and summarize the evidence from studies that examined the mental health of older adults with DSL. In accordance with the Preferred Reporting Items for Systematic Reviews (PRISMA) statement, specific databases were searched and eight articles were selected for final review. Seven studies investigated the association between DSL and depression or depressive symptoms, whilst one study explored the relationship between DSL and quality of life. No studies investigated the impact of DSL on anxiety. Overall, results of this review suggested that there is a significant relationship between DSL and decreased mental health with those with DSL either displaying depressive symptoms or being at risk for developing depression. Future research should focus on comparative studies of older people with and without sensory loss, as well as targeted studies of older people with dual sensory loss, that incorporate well-defined and valid measures of sensory loss and mental health.

## Background

Mental health is a key component in the health and well-being of older adults and refers to the “state of well-being in which every individual realizes his or her own potential, can cope with the normal stresses of life, can work productively and fruitfully, and is able to make a contribution to her or his community” (World Health Organisation, 2013b, para. 1). Dual sensory loss (DSL) is the combined loss of vision and hearing and is a common contributor to the mental health and well-being of older adults (Heine and Browning, 2002).

DSL is particularly prevalent in the older adult population due to the gradual deterioration of their vision and/or hearing with advancing age (Davila et al., 2009), and particularly for veterans who are aged 85 years or older (Smith et al., 2008). In a Danish study of DSL rehabilitation clients Dammeyer (2013) found that DSL increased rapidly in those aged 65 years and over. In a similar Canadian study Wittich et al. (2012) found that 69% of DSL rehabilitation clients were aged 65 years and over. According to Caban et al. (2005) DSL increased from 1.3% for 18 to 44 year olds to 6.6% in those aged 80 years and older. Schneider et al. (2012) estimated that 25% of participants aged 80 years and over in the Australian Blue Mountains Eye Study experienced combined vision and hearing loss. In a European study of older people aged 50 years and over 5.9% reported DSL (Viljanen et al., 2013). The significance and potential impact of DSL in older age groups was highlighted by Brennan and Bally (2007) who estimated the prevalence of DSL in those aged 70 years and over as ranging between 5 and 20%. They concluded that by 2030, based on population ageing trends, between 3.5 and 14 million older people in the US would develop DSL.

DSL is an acquired condition, which gradually deteriorates over time. DSL is poorly understood (Davila et al., 2009), under-recognized and under-diagnosed (Heine et al., 2002). This is particularly the case in its mild form since it may be undetected by the individual, or onset may initially be in one domain at a time (vision or hearing deterioration). Older adults with DSL are more likely to have health problems, reduced activities and restricted social roles as compared to those with no sensory loss or unisensory loss (Crews and Campbell, 2004). The consequences of DSL (even mild DSL) are significant and include psychosocial difficulties, withdrawal from communication-based situations (Heine et al., 2002), avoidance of social interactions and diminished quality of life (Dalton et al., 2003; Brennan et al., 2006).

The World Health Organization estimates that 20% of adults aged 60 years and over suffer from a mental or neurological disorder (World Health Organisation, 2013a). The most common mental health disorders in older adults are dementia, depression and anxiety. Approximately 7% of older people worldwide suffer from unipolar depression and about 3.8% of older people suffer from anxiety disorders (World Health Organisation, 2013a). Depression is a serious mental health disorder that influences quality of life and although it is known to be associated with DSL, few researchers have investigated its association and impact. In a cross sectional study of adults aged over 55 years, Capella-McDonnall (2005) estimated that of the 7.3% of the sample who experienced DSL, 35% experienced depression suggesting a significant relationship between DSL and depression. This finding has been corroborated by other researchers such as Chou and Chi (2004), Chou (2008), and Harada et al. (2008).

The literature investigating DSL in older adults is limited, although numerous studies have been published investigating a wide range of other health issues in ageing populations. A contributing factor to the paucity of research on DSL in older people is that DSL is a multisensory disorder, and requires the collaboration of various diverse professional disciplines to assess and manage the disorder. Public awareness of DSL is also not widespread and in turn, rehabilitation options have not been well-explored in many countries. Exceptions to this are discussed in papers emerging from the US, Canada and Australia (Heine and Browning, 2002; Saunders and Echt, 2007, 2012; Wittich et al., 2012).

To date, no systematic reviews of the relationship between mental health and DSL have been conducted. A review of the research is thus timely so that the association between DSL and mental health issues in older adults can be identified and used to inform programs and policies to promote healthy ageing in older people. A systematic review of the literature can also provide health professionals with evidence-based knowledge about the impacts of DSL in older adults and assist them in designing programs that will contribute to improving health outcomes in their older clients.

The aim of this systematic review was to critically review and summarize the evidence from studies that examined DSL and mental health issues (in particular depression, anxiety, and well-being) in older adults and evaluate the quality of the evidence by comparing it to six areas of the STrengthening the Reporting of Observational studies in Epidemiology (STROBE) statement (University of Bern, 2009).

## Methods

### Evidence acquisition

#### Literature search

In accordance with Preferred Reporting Items for Systematic Reviews (PRISMA) recommendations (Moher et al., 2009), from March—November 2012, the databases Medline, PsycINFO, Cochrane Library, Sociological abstracts and Scopus were searched for relevant studies (articles from 1990 onwards were searched). The search was updated in January 2013. Groups of thesaurus terms as well as free terms were used to search the databases. Terms for *older adults* (thesaurus terms OR elderly, seniors, aging or ageing) were used in AND-combination with each of the terms *vision and hearing loss, vision and hearing impairment, DSL or dual sensory impairment*. Thereafter studies were searched for the terms *mental health, anxiety, and depression*. Additional articles were identified by manually searching known articles in the area of DSL.

#### Inclusion criteria and selection process

In order to be included in the review, studies were required to meet the following criteria:

1. Focused on older adults (included adults at least 60 years and above);
2. Reported vision and hearing loss as a combined construct; and
3. Focused on an aspect of mental health.

Only full-text, peer-reviewed articles written in English were considered for inclusion. Titles, keywords, and abstracts of articles identified through the search process were reviewed to identify eligible papers. The first author initially checked eligible papers to exclude articles that were out of the scope of this study. Subsequently, the first and second author independently reviewed all potentially relevant references for eligibility. Disagreements between these reviewers were discussed with a third person (a research assistant) and a consensus decision was made.

### Data extraction and quality assessment

The first author extracted data on the study population. On the basis of the design and methodology of individual studies, quality descriptors and ratings were derived using the STROBE statement appraisal system. The STROBE statement includes six areas for evaluation that need to be considered when appraising research, namely, the title and abstract, introduction (background/rationale and objectives), methods (study design, setting, participants, variables, data sources/measurement, bias, study size, quantitative variables and statistical methods), results (participants, descriptive data, outcome data, main results, and other analyses), discussion (key results, limitations, interpretation, generalizability) and other information (such as funding). Each study was appraised according to these areas.

### Evidence synthesis

#### Study selection

**Figure 1:**
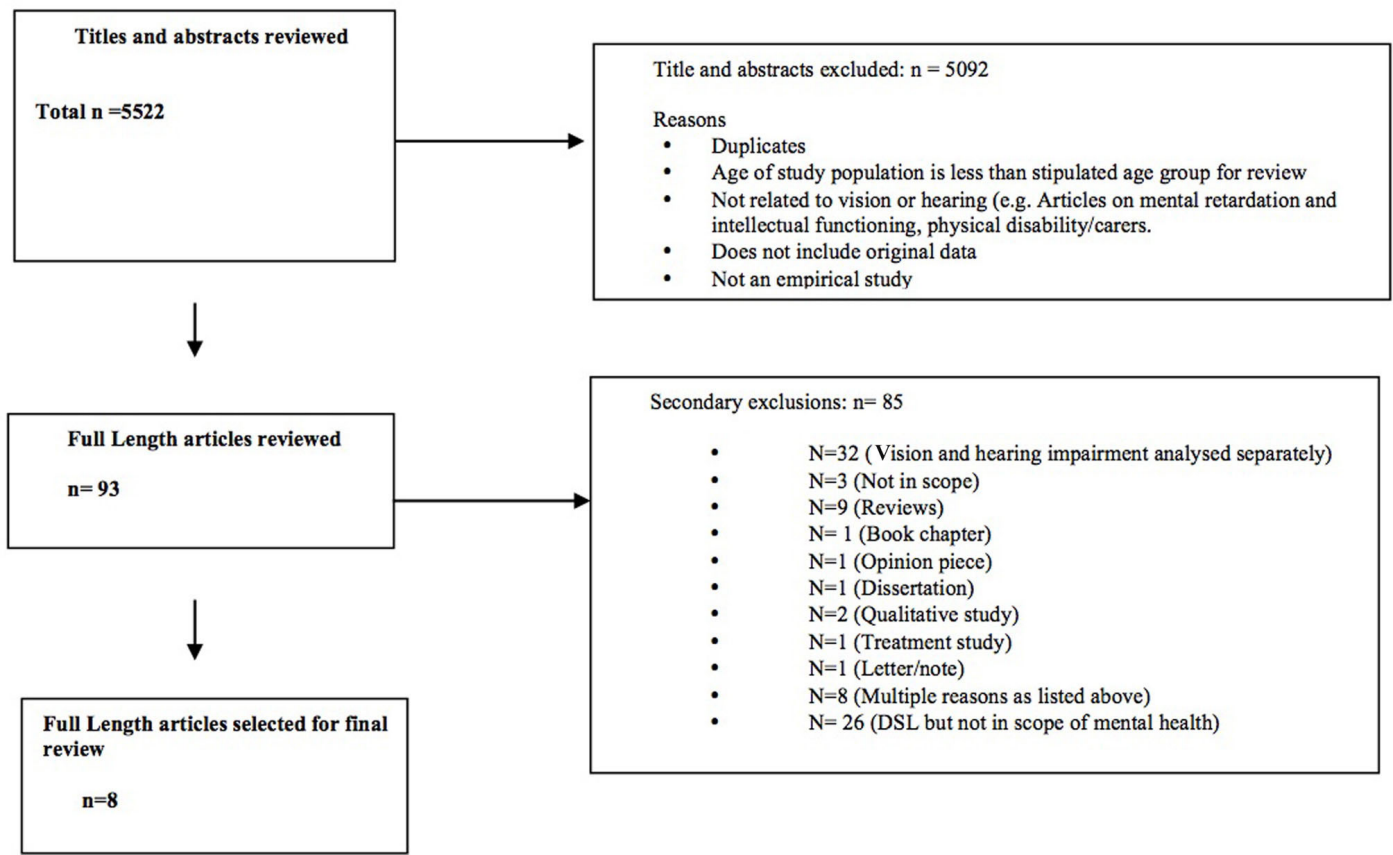
**Article selection process**.

The literature searches yielded 5522 unique potentially relevant articles (see Figure 1). A total of 5092 articles were classified as out of scope due to: duplication of articles, the sample did not include people aged 60 years and over (for example, included younger people with deaf-blindness due to Ushers Syndrome), the study did not focus on sensory loss, the study did not include original data and the article was not based on an empirical study. After excluding the records out of scope, the full text of each of the remaining 886 records was checked. In total, 793 (89%) of these 886 articles did not meet the inclusion criteria. The most common reasons for primary exclusion were that the article focused only on vision impairment (*n* = 489, 55.2%) or hearing impairment alone (*n* = 261, 29.4%). The 93 relevant articles that remained were subject to secondary screening. The most common reasons for secondary exclusion were the focus in the results section on separate sensory losses (*n* = 29, 31.2%) rather than a combined dual sensory construct or a focus on DSL but not on mental health impacts (*n* = 26, 27.9%) Finally, eight studies were included in this review. Figure 1 shows the study selection process and Table 1 summarizes the studies included in the review.

**Table 1:** **Summary of reviewed articles**.

**References**	**Aim**	**Design**	**Age group**	**Measurement instruments**	**Results**
Capella-McDonnall, 2005	To determine the effect of DSL on depressive symptoms and whether people with DSL were more likely than those with a single sensory loss to experience depressive symptoms.	Cross-sectional. Secondary analysis of the 2001 National Health Interview Survey.	55 years and over	Questionnaire: depressive symptoms, health education, poverty, social activities and social support and functional disability (ADL and IADL status).	Prevalence: 7.3% had DSL, 8.9% had VL only, 24.9% had HL only, and 58.9% = NSL.
	Terms used: DSL, Dual Sensory Loss; VL, Vision Loss; HL, Hearing Loss; NSL, No sensory loss	Community dwelling adults in US. Logistic regression.		Sensory assessment:Vision—Do you have trouble seeing even when wearing contact lenses or glasses? Hearing—4 point scale for hearing without a hearing aid	Proportion of people experiencing depression were: DSL—0.35; VL—0.28; HL—0.19; NSL—0.14. There was a relationship between all types of sensory loss and symptoms of depression. People with DSL were more likely to experience symptoms of depression.
		*N* = 9832 (unadjusted analysis) *N* = 6089 (analysis in this study using control variables)			This relationship was lower but still significant after controlling for covariates. Those with DSL were not significantly more likely than those with VL to experience depressive symptoms, but were significantly more likely than those with HL to experience symptoms of depression
Chia et al., 2006	To assess the relationship between hearing and vision impairment and the impact of vision, hearing, and dual sensory loss on quality of life. Terms used: Hearing impairment, Vision impairment, Combined sensory impairments	Population based cohort study: Blue Mountains Eye Study.	55–98 years	Questionnaire: SF-36	Those with both hearing and vision impairments were more likely to show lower SF36 scores compared to those with either vision or hearing impairment. This relationship occurred for both the physical and mental health component scores of the SF36
		5 year follow up of baseline data included measurement of hearing impairment.		Sensory assessment:Vision—Monocular distance logMAR visual acuity using retroilluminated chart. Visual impairment defined as visual acuity less than 20/40 in the better eye.	
		Logistic regression used to examine impact of sensory loss on quality of life.		Hearing—Pure tone air-conduction audiometry. Hearing impairment defined as pure tone average air-conduction threshold worse than 25-dB	
		*N* = 2015			
Chou and Chi, 2004	To assess the relationship between the combined effect of visual and hearing impairment on depressive symptoms amongst Chinese older adults.	Cross-sectional. Representative sample of community dwelling Chinese older people living in Hong Kong.	60 years and over	Face-to-face interview: Socio-demographic variables, health indicators (self-rated health and health conditions), ADLs and IADLs, family support, and depression (15-item Geriatric Depression Scale).	Prevalence: 20% had vision impairment, 17.5% had hearing impairment, and 6.5% had dual sensory impairment.
	To examine the relationship between sensory impairment and depressive symptoms after controlling for variables related to functional capacity. Terms Used: Vision Impairment, Hearing Impairment, Double sensory impairment	Four logistic regression models were performed to assess the impact of sensory impairment on depression.		Sensory assessment: Questionnaire—rate vision and hearing with aids on a 4 point scale: 1 = very good; 2 = good; 3 = poor; 4 = almost or completely unable to see or hear. Two binary variables created for visual and hearing impairment, respectively, (good and very good = 0 and poor and almost or completely unable to see or hear = 1)	Vision impairment was significantly related to depression even after age, gender, marital status, education, self-reported health status, the presence of 11 diseases, functional limitations and family support were controlled but hearing loss was not significantly related to depression. Hearing impairment did not add to the likelihood of depression when vision impairment was already present
		*N* = 2003			
Chou, 2008	To examine the role of dual sensory loss in the onset and persistence of depression in older persons living in the UK.	Longitudinal, (2-year), prospective, observational, population based study as part of the English Longitudinal Study of Ageing (ELSA) Waves 1 and 2 conducted in the UK. Evaluation at 2 time points 24 months apart.	65 years and over	Questionnaire: 8-item CESD, socio- economic variables, health indicators (medical conditions), functional health (mobility ADLs and IADLs), health behaviors (smoking and alcohol consumption) and social support.	Vision loss predicted both the onset and persistence of depression even after covariates adjustment. The association between dual sensory loss and depression did not remain once health indicators were controlled for
	Terms used: Visual impairment, Hearing impairment, Both visual and hearing impairment, Dual Loss	Odds Ratios and Logistic regression were used to analyse data.		Sensory assessment: Rating scale with aids.	
		*N* = 3782		Vision—6-point rating scale (ranging from excellent to legally blind).	
				Hearing—5-point rating scale (ranging from excellent to poor).	
				Two binary measures of sensory impairment created to indicate poor eye sight (0 = excellent, very good, and good; 1 = fair, poor, and legally blind) and poor hearing (0 = excellent, very good, and good; 1 = fair and poor)	
Harada et al., 2008	To evaluate the association of hearing impairment, vision impairment, and their combination (dual sensory impairment) with depression, self rated health and functional activity in community-dwelling older adults, and to examine gender effects.	Cross-sectional study conducted in a rural Japanese town (Kurabuchi Town, Takasaki City, Gunma Prefecture—rural village with a population of approximately 4800). Multiple logistic regression analyses used to analyse data.	65 years and over	Questionnaire and interview (home visit): Assessed depression (using the Geriatric Depression Scale), Self rated health, and functional activity (using the Tokyo Metropolitan Institute of Gerontology's Index of Competence).	DSI associated with depression, poor self rated health and reduced functional activity in both men and women
	Terms used: Vision impairment only (VIO), Hearing impairment only (HIO), Dual Sensory impairment (DSI), No sensory impairment (NSI)	*N* = 843 (351 males, 492 females)		Sensory assessments: Objective examination. Vision—best corrected visual acuity using the Landolt broken ring chart at 5 m. Impairment = acuity of worse than 0.5 in the better eye.	
				Hearing—pure-tone test conducted in a separate quiet room. Both ears tested separately at 30 dB at 1 kHz and 40 dB at 4 kHz. Impairment = fail to hear 30-dB signal at 1 kHz bilaterally.	
				DSI = participants with both vision and hearing impairment.	
				Participants categorized into DSI, VIO, HIO, or NSI	
Lupsakko et al., 2002	To investigate the association between functional sensory impairment and depressive symptoms and depression diagnosed according to the DSM-IV criteria.	Cross sectional.	75 years and over	Interview, questionnaire and DSM-IV criteria.	Prevalence of depression in CSI group = 18% compared to 15% ASF group. The differences between these groups were insignificant.
	Terms used: Functional Visual impairment (FVI), Functional hearing impairment (FHI), Combined functional sensory impairment (CSI), Adequate Sensory Function (ASF)	Population-based sample. Logistic regression;		Depression—DSM-IV checklist; and Zung Depression Status Inventory (cut off points of 40/80 and 48/80 used).	The difference in depression between the ASF group and sensory impairment groups (including FHI, FVI, and CSI groups) was statistically significant.
		Kolmogorov–Smirnov statistics, with a Lilliefors significance or Shapiro–Wilk statistic; and correlation coefficients (Spearman method).		Sensory assessment: Objective for vision and observation for hearing.	Depressive symptoms, but not major depression, were common if older persons had combined sensory impairment
		*N* = 470		Vision—Snellen eye charts with E-letters and reading charts using the Finnish Center for Visually Impaired test.	
				FVI = binocular visual acuity, either for near or distance vision of less than 20/60. Hearing—The ability to conduct a face-to-face conversation, hearing aid use and self-reported hearing problems.	
				FHI = if person had clear difficulty with conversation due to poor hearing acuity; the person expressed hearing as a main health problem; had difficulty hearing or had earlier ordered a hearing aid.	
				CSI = those with combined functional sensory impairment	
McDonnall, 2009a	To determine the effect of developing a dual sensory loss (DSL) on depression over time and evaluate the impact of pre-existing single sensory loss on this effect.	Longitudinal study.	38–95 years (DSL Group).	Questionnaire: Demographics (minority status, gender, age) and CES-D depression scale.	Descriptive—Almost two thirds of the DSL sample experienced one sensory loss prior to the DSL. The remaining 35% reported both sensory losses at the same time point.
	Terms used: Hearing loss, Vision loss, Dual Sensory Loss (DSL)	Data obtained from the Health and Retirement Study (HRS) and the Aging and Health Dynamics study (AHEAD). Two groups: (a) those who developed DSL during the study and did not at a later time report improved hearing or vision (DSL group), and (b) equal number of persons who did not report sensory loss during the study, matched to the DSL group on the basis of age and gender (comparison group).	40–93 years (Comparison Group)	Sensory assessment: Questionnaire.	A significant increase in depression at onset of DSL. Depression increased at following DSL onset.
		Multilevel modeling, trajectories.		Vision – rating scale—“With your glasses, is your eyesight excellent, very good, good, fair, or poor?” and if legally blind (volunteered information).	Those who developed DSL showed higher depression scores at the start of study than those with no sensory loss
		*N* = 1380 (with DSL)		Impairment = report of fair or poor eyesight; or legal blindness. Hearing- rating scale—“With your hearing aid, is your hearing excellent, very good, good, fair, or poor?” Impairment = report of fair or poor hearing. DSL = reports of both vision loss and hearing loss at the same time point. (Vision loss and hearing loss modeled as time-invariant variables, measured at the time point prior to the report of DSL)	
McDonnall, 2009b	To identify risk factors associated with depression among older adults with dual sensory loss.	Cross sectional.	55 years+	Questionnaire: Demographics, CES-D depression scale, activity (functional disability, IADLs, physical activity and activity loss) and social factors (social support).	Worsening vision and worsening hearing reported separately in relation to the association with depression. In the final regression model, 39.6% of the variance in depression was due to dual sensory loss
	Terms used: Vision loss, Hearing loss, Dual sensory loss	Small part of a larger study (The Persons Aging with Hearing and Vision Loss study, PAHVL). Data obtained through surveys from two groups. Primary data = group of older adults with sensory loss. Secondary data = a nationally representative sample of older adults (PAHVL study).		Sensory assessment: Questionnaire. Vision—worsening vision (is vision getting worse?)	
		Correlation and hierarchical linear regression.		Hearing—worsening hearing (is hearing getting worse?)	
		*N* = 203		Sensory loss—difficulty with communication, difficulty with functional activities due to sensory losses	

## Results and discussion

The eight reviewed studies were analyzed according to the STROBE statement.

The following results were obtained:

### Rationale and objectives

The majority of the studies reviewed (*n* = 7) aimed to investigate the association between sensory loss (including vision loss, hearing loss and DSL) and depression or depressive symptoms. Harada et al. (2008) evaluated the impact of additional variables such as self rated health and functional ability. One study focussed specifically on the impact of hearing impairment, vision impairment and DSL on quality of life as measured by the SF36 (Chia et al., 2006). Overall, all eight studies adequately described the aims of the study.

### Methods

#### Study population

Overall, there were five studies that only included older participants (defined as 60 years and over for the purposes of this review) and reported the results in a way that enabled specific conclusions to be drawn about DSL in older groups (Lupsakko et al., 2002; Chou and Chi, 2004; Chia et al., 2006; Chou, 2008; Harada et al., 2008). The remaining three studies (Capella-McDonnall, 2005; McDonnall, 2009a,b) included both young and old participants. These studies examined the effects of DSL in the full sample and did not differentiate between younger and older age groups in the analyses.

#### Sample size

The sample size in the selected studies ranged from 203 participants to samples in which data was primarily derived from large population-based studies that included up to 9832 participants (although not all were in the older adult age range or had DSL since they were primarily prevalence studies of, for example, age related conditions). For example, in the Capella-McDonnall (2005) study, although there were 9832 participants in the unadjusted analysis and subsequently 6089 participants in the adjusted analyses, only 7.3% had DSL. Overall the sample sizes of the studies were adequate for the types of analyses performed in the respective studies.

#### Research design

A variety of methods were used to investigate DSL, however cross-sectional studies were the most common (*n* = 6). There were two longitudinal studies (Chou, 2008; McDonnall, 2009a). Chou (2008) used 2-year prospective data from the English Longitudinal Study of Ageing and McDonnall (2009a) used data from 9 waves of the US Health and Retirement Study. Chia et al. (2006) examined 5-year vision and hearing impairment follow up data from a baseline study of vision impairment. Two studies were designed specifically to investigate the impacts of DSL in older people (Chou and Chi, 2004; Harada et al., 2008) while the remaining studies were based on larger general studies of older people including the US Health and Retirement Study, the English Longitudinal Study on Aging, the US National Health Interview Survey, the Finnish Kuopio 75+ study and the Australian Blue Mountains Eye Study.

#### Recruitment strategy

All studies achieved well on this criterion. Participants in reviewed studies were mostly community dwelling, accessed via door-to-door interview, telephone or at senior centers. Only one study was conducted in a rural population (Harada et al., 2008). Countries were well-represented with three studies conducted in the US (Capella-McDonnall, 2005; McDonnall, 2009a,b) and one study in each of the following countries: Japan (Harada et al., 2008), Finland (Lupsakko et al., 2002), Hong Kong (Chou and Chi, 1999), UK (Chou, 2008), and Australia (Chia et al., 2006). All studies included men and women however no studies specifically reported results for any minority ethnic groups within the study country.

#### Data collection—measurement of sensory loss and mental health variables

There were varied methods used to obtain information relating to vision and hearing loss. Primarily, self-reported difficulty, or completion of a rating scale, were used to evaluate the presence of sensory loss. There were six studies that evaluated self-reported vision and self-reported hearing difficulty (Lupsakko et al., 2002; Chou and Chi, 2004; Capella-McDonnall, 2005; Chou, 2008; McDonnall, 2009a,b) with one study (Lupsakko et al., 2002) using observations of the ability to conduct a face-to-face conversation, hearing aid use and self-reported hearing problems. Objective measures (primarily the Snellen visual acuity test) were used to evaluate visual acuity in two studies (Lupsakko et al., 2002; Chia et al., 2006). Pure-tone audiometry was used in two studies to measure hearing acuity (Chia et al., 2006; Harada et al., 2008).

Measurement of mental health also varied across the studies. Seven of the studies either measured depression or the occurrence of depressive symptoms. One study (Capella-McDonnall, 2005) used a questionnaire to evaluate depressive symptoms, whilst six studies used a standardized scale to evaluate depression. Most commonly, the CES-D was used (Chou, 2008; McDonnall, 2009a,b) with the Geriatric Depression Scale (Chou and Chi, 2004; Harada et al., 2008) also being used. One study used two measurements to evaluate mental health. Lupsakko et al. (2002) utilized the DSM-IV checklist and Zung Depression Status Inventory. Chia et al. (2006) measured quality of life using the SF36 but not depression. In all studies, data collection methods were well-described.

#### Data analysis

Multiple methods were used to analyse the data including descriptive and inferential statistics such as multivariate analysis. Most commonly, regression analysis was used to examine the relationships between sensory impairment and mental health (Lupsakko et al., 2002; Chou and Chi, 2004; Capella-McDonnall, 2005; Chia et al., 2006; Chou, 2008; Harada et al., 2008; McDonnall, 2009a,b). Multilevel modeling was used to show the effects of developing DSL over time in one study (McDonnall, 2009a).

### Results and discussion of reviewed studies: findings

Overall all studies provided a clear statement of findings relevant to this review. In relation to DSL, Capella-McDonnall (2005) estimated that out of a total sample of 6089 participants, 7.3% had DSL of whom 35% had depression. Although the prevalence of DSL was consistent with the findings of Chou and Chi (2004) who found that 6.5% of their sample (community dwelling, Chinese participants aged 60 years and over) had DSL, these later authors found that vision impairment was significantly related to depression whilst hearing loss was not. Furthermore, Harada et al. (2008) found that DSL was also associated with poor health and reduced functional activity. DSL was associated with lower scores on both the mental health and physical health component scores of the SF36 compared to those with hearing or vision impairment (Chia et al., 2006). In relation to the development of depression and depressive symptoms, McDonnall (2009a) found a significant increase in depression at the first report of DSL, with depression increasing at a significantly faster rate following DSL. However, participants adjusted to the impairments over time. Lupsakko et al. (2002) concluded that depressive symptoms, but not major depression, are common in older people with DSL and McDonnall (2009b) found that almost 40% of the variation in depression was due to DSL.

## Conclusion

The aims of this systematic review were to critically review and summarize the evidence from studies that examined the mental health of older adults with DSL and evaluate the quality of the evidence by comparing it to the STROBE statement. Most of the studies reviewed provided clear aims for their study, collected their data appropriately, described the method and data analysis well, provided valid findings and were applicable to this review and therefore contributed significantly to the evidence base concerning DSL and mental health in older adults. However, some studies that included younger adults did not differentiate the effects of DSL between different age groups.

Evaluation of the studies revealed that the selected studies were primarily cross-sectional in nature and based on large population-based studies not specifically designed to investigate the association between DSL and mental health in older people. Studies primarily collected sensory impairment data via self-report and less commonly by standardized vision and hearing acuity measurement. Depression, measured by a standardized depression scale, was the most common mental health variable investigated. Well-being was less frequently explored and there were no studies that investigated anxiety in participants with DSL.

In order to better understand the impacts of DSL on mental health in older people future research should address a number of methodological issues. It is important that future studies use both objective and subjective measures of DSL. A related issue is the definition of DSL. Studies vary in impairment cut off points for both hearing and visual acuity and while there is no obvious gold standard we would welcome further discussion and consensus on this issue. With increasing longevity and the heterogeneity of the ageing process future studies should include samples across the full older age. Very little is known for example about the impact of DSL in people in their eighties and nineties where other significant co-morbidities (including cognitive impairment) may interact with sensory loss to impact mental health. Comparative studies focussing on the relative impact of no sensory loss, single and DSL are needed to explore whether sensory loss has additive effects or whether older people adapt to multiple losses. While longitudinal studies are expensive, such approaches are important for understanding more fully the causal relationships between DSL and mental health outcomes in old age. Finally while a focus on the impact of DSL on depression is important other mental health problems such as anxiety should be included in future studies. Anxiety is under–researched in the older population even though there is now evidence that anxiety is more prevalent than depression in community samples of older adults (Bryant et al., 2013).

## Author contributions

Both authors conceptualized and designed the review. Author A drafted the paper and Author B revised the paper. Both authors have final approval of the published article and both authors agree to be accountable for all aspects of the work in ensuring that questions related to the accuracy or integrity of any part of the work are appropriately investigated and resolved.

### Conflict of interest statement

The authors declare that the research was conducted in the absence of any commercial or financial relationships that could be construed as a potential conflict of interest.
